# m6A Methyltransferase 3 Promotes the Proliferation and Migration of Gastric Cancer Cells through the m6A Modification of YAP1

**DOI:** 10.1155/2021/8875424

**Published:** 2021-08-04

**Authors:** Wenjie Zhou, Qingying Xian, Qi Wang, Chen Wu, Haijiao Yan, Xiaodong Li, Lu Lu, Changping Wu, Danxia Zhu, Xiaoli Xu, Jun Wu

**Affiliations:** ^1^Department of Oncology, The Third Affiliated Hospital of Soochow University, 185 Juqian Street, Changzhou, Jiangsu Province, China; ^2^Department of Geriatrics, The Third Affiliated Hospital of Soochow University, 185 Juqian Street, Changzhou, Jiangsu Province, China

## Abstract

Gastric cancer is the most common gastrointestinal tumor with an increasing incidence. Furthermore, advanced gastric cancer is more common, but the mechanism underlying the proliferation and metastasis of gastric cancer has not been thoroughly explored. N6-methyladenosine (m6A) methyltransferase 3 (METTL3) may be involved in the proliferation and metastasis of gastric cancer. Therefore, Yes-associated protein 1 (YAP1) in the Hippo pathway was selected as the target, and the relationship between METTL3 and the proliferation and metastasis of gastric cancer was proved through a series of experiments. This research showed that the expression of m6A and METTL3 was upregulated in human gastric cancer tissues and gastric cancer cell lines. After lentiviral transfection, *METTL3* silencing in AGS (human gastric adenocarcinoma cell line AGS) and MKN-45 (human gastric cancer cell line MKN-45) gastric cancer cell lines directly inhibited the proliferation, aggressiveness, and migration of gastric cancer cells. Mechanically, the inhibition of the YAP1-TEAD signaling pathway by peptide 17 reduces m6A methylation and the total mRNA level of YAP1. It also eliminates the promoting effect of *METTL3* on the proliferation and migration of gastric cancer cells. In turn, the overexpression of *YAP1* eliminates the inhibitory effect of *METTL3* silencing on the proliferation, migration, and invasion of gastric cancer cells. This article proved that m6A methyltransferase METTL3 promoted the proliferation and migration of gastric cancer cells through the m6A modification of YAP1.

## 1. Introduction

Gastric cancer has become the most common gastrointestinal tumor with increased morbidity and mortality, ranking fourth in the global incidence of cancer [1]. Gastric cancer is the result of a combination of many factors. Some relevant studies have suggested a relation of gastric cancer with the malignant transformation of *Helicobacter pylori*, environment, genes, high-salt diet, and even stem cells on the mucosal surface of gastric cancer cells [2]. Early gastric cancer is more difficult to diagnose, and therefore, the proportion of patients with advanced gastric cancer is still high [3]. It is now generally believed that *H. pylori* is the main cause of gastric cancer. Hence, it is more apt to say that the inflammatory response induced by *H. pylori* directly leads to DNA methylation, but not all kinds of inflammation induce abnormalities in DNA. Abnormal DNA methylation is caused by specific inflammation and may be related to the expression of interleukin-1beta (Il1b), nitric oxide synthase-2 (Nos2), and tumor necrosis factor (Tnf), while the inactivation of tumor suppressor genes, such as *P16*, *hMLH1*, and *CDH1*, and the activation of the Wnt (wingless/integrated) pathway may also be caused by abnormal DNA methylation [4]. The progression of gastric cancer is also closely related to the imbalance of methylation. Studies have shown that these abnormal methylation modifications caused by the imbalance of methyltransferase are usually reversible [5], thus providing a potential target for therapy and helping to improve the prognosis of gastric cancer.

More than 100 chemical modification methods are available for mRNAs [6]. N6-methyladenosine (m6A) is the most common internal modification in higher eukaryotic mRNAs. It refers to the addition of a methyl group at the position of adenosine N6. According to statistics, on average, about 0.1%–0.4% of adenosine in mRNAs is modified by m6A [7]. The regulator of m6A is also important in the proliferation, metastasis, and invasion of cancer [8]. The regulator of m6A is also important in the proliferation, metastasis, and invasion of cancer [9], which are found near termination codons, 3′-untranslated regions (3′-UTRs), and long internal exons [10, 11]. M6A is catalyzed by a multicomponent methyltransferase complex [12]. The m6A methyltransferase complex mainly includes methyltransferase 3 (METTL3), methyltransferase 14 (METTL14), and Wilms tumor 1-related proteins (WTAP), where METTL3 serves as the catalytic core. METTL14 serves as the structural support for RNA binding, and WTAP serves as an adaptor protein for the interaction between METTL3 and METTL14, thereby significantly affecting the m6A load in the cells [13]. In addition, the complex also includes proteins such as vir-like m6A methyltransferase (VIRMA, also known as KIAA1429) and RNA-binding motif protein-15 (RBM15) [14]. The m6A methyltransferase complex can catalyze the transfer of methyl groups from the donor substrate S-adenosylmethionine to adenine nucleotides in the acceptor RNA substrate [15]. After m6A is formed, it can also be reversed to mRNA by alkylation repair homolog protein 5 (ALKBH5) and fat-mass- and obesity-related protein (FTO) by RNA demethylase in mammals, thus maintaining the homeostasis of mRNA methylation and demethylation [16].

METTL3 is a key component of the large m6A methyltransferase complex in mammals and is responsible for modifying m6A in various RNAs [17]. Some studies claim that, besides methyltransferase activity, the catalytic subunit of METTL3 can help increase the level of elF3 to promote cancer gene translation. Mechanistically, METTL3 promotes the translation of a subset of mRNAs with m6A peaks near the stop codon [18]. That is to say, METTL3 enhances translation only when tied to the report gene near the termination codon [19]. Many recent studies have confirmed the involvement of METTL3 in various physiological mechanisms, pathological processes, and even the occurrence and development of tumors. METTL3 is a potential target favored by many scholars, but its proliferation and the mechanism of metastasis are not yet clear. By reading the literature, we determined that YAP1 is the downstream target of METTL3 [20, 21] and YAP1 is the core effector in the Hippo pathway, which can control cell proliferation and differentiation [22, 23]. This study showed that METTL3 promoted the proliferation and metastasis of gastric cancer by modifying Yes-associated protein 1 (YAP1) mRNA m6A.

## 2. Materials and Methods

### 2.1. Patient and Clinical Samples with Ethical Approval

A total of 40 pairs of patient specimens were used in this study. These human samples were obtained according to the principles of the Declaration of Helsinki and approved by the Third Affiliated Hospital of Soochow University (Jiangsu, China). A written informed consent form was obtained from all patients.

### 2.2. Culture of Human Normal Gastric Epithelial Cell Line and Gastric Cancer Cell Line

Human normal gastric mucosal epithelial cell lines (GES-1) and gastric cancer cell lines (AGS, MKN-45, and MKN-28) were cultured in DMEM containing 10% fetal bovine serum. The culture medium was changed daily and passaged every 3 days.

### 2.3. M6A RNA Methylation Quantification

Poly A + RNA purification and quantification: the cells were spotted on a nylon membrane after denaturation, air-dried, cross-linked using ultraviolet light, sealed with skimmed milk, incubated with m6A antibody overnight, washed the next day, incubated with secondary antibody at room temperature for 1 h, and imaged by chemiluminescence. An m6A RNA Methylation Assay Kit (Abcam, ab185912, Epigentek Group, Farmingdale, NY) was used to evaluate the content of m6A in total RNA.

### 2.4. Quantitative Polymerase Chain Reaction (qPCR)

Total RNA was cleaved using TRIzol reagent and used to synthesize cDNA using a one-step RT-PCR kit (Thermo Fisher Scientific, CA). An ABI Vii7 system (Applied Biosystems, USA) was used for real-time PCR. Glyceraldehyde-phosphate dehydrogenase (GAPDH) was used as a housekeeping gene. The relative gene expression level was calculated by the comparative CT method (ΔΔCT). The primers used in the study are listed in Table 1.

### 2.5. Western Blot Analysis

Western blot analysis was performed with antibodies against METTL3, YAP1, and GAPDH (Abcam, MA, USA) as previously described. Human GAPDH was used as an endogenous control to normalize the protein loading.

### 2.6. Cell Proliferation Assay

A cell counting kit-8 (CCK-8, Beyotime, Shanghai, China) was used to measure cell proliferation.

### 2.7. Colony Formation Assay

The cells were counted and seeded in Petri dishes. The cell density in each well was 500, and the cells were cultured for 14 days until colonies were visible. The cells were washed with phosphate-buffered saline and fixed with paraformaldehyde for 15 min. The colonies were stained with Giemsa's solution for 15 min and washed with tap water. After air drying, colonies with more than 50 cells were counted.

### 2.8. Migration and Invasion Assay

Cell scratch assay was used to detect the migration distance of cells before (0 h) and 24 h after the scratching of inoculated cells. The transwell (without Matrigel) assay was used to detect the migration of gastric cancer cells 24 h after cell inoculation. A Transwell chamber (8 mm hole, BD Falcon, NJ, USA) was used to detect gastric cancer cell invasion 24 h after cell inoculation.

### 2.9. The Methylated m6A RNA Immunoprecipitation (m6A Me-RIP) Assay

According to literature methods [24], the methylated m6A RNA immunoprecipitation (me-RIP) was performed as described. Poly A + RNA was isolated, purified, and precipitated with an m6A antibody (Abcam, MA, USA). The methylated RNA was subjected to qPCR analysis for methylated YAP1 mRNA level.

### 2.10. Cell Viability Assay

The viability of HK-1 cells was detected using a Cell Counting Kit 8 (Beyotime, Shanghai, China).

### 2.11. TEAD-Dependent Luciferase Activity

For TEAD-dependent luciferase activity assays, the indicated HK-2 cells were transfected with 200 ng pGL3-TEAD reporter and 10 ng pRL-TK (Promega) using Lipofectamine 3000 reagent (Thermo Fisher Scientific, CA). Luciferase activity was then assayed using a dual-luciferase reporter assay system (Promega).

### 2.12. Establishment of the Metastatic Gastric Cancer Tumor Model

A gastric cancer model was established by injecting gastric cancer cells into the skin of each nude mouse (12 nude mice in total). We observed the growth of the tumors, and the tumor size was measured at D0, D3, D6, D9, D12, D15, D18, D21, and D24 (the observation time is 24 days). One month after the injection, the subcutaneous tumors of nude mice were excised, and immunohistochemical tests were performed.

### 2.13. Immunohistochemistry

The tissue sections were blocked with 5% Bovine Serum Albumin (BSA) and then incubated with a rabbit anti-human METTL3 antibody at 1 : 200 dilution and 4°C overnight. Finally, the tissue section was incubated with horseradish peroxidase-labeled (HRP-labeled) goat anti-rabbit secondary antibody, followed by incubation with diaminobenzene as the chromogen and hematoxylin as the nuclear counterstain.

### 2.14. Statistical Analysis

Data were expressed as the means ± SEM. The unpaired, 2-tailed *t*-test was used for comparisons between 2 groups. For multiple comparisons, analysis of variance (ANOVA) or repeated ANOVA followed by the Bonferroni post hoc test was used with GraphPad Prism®version 6.0 software. The *P* value <0.05 was considered statistically significant.

## 3. Results

### 3.1. Difference in the Expression of m6A and METTL3 in Gastric Cancer Tissues and Adjacent Tissues

**Figure 1:**
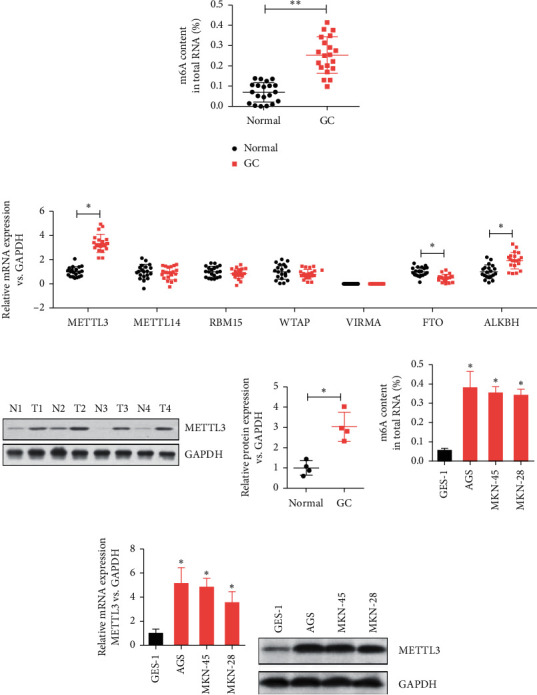
Levels of m6A methylated RNA and METTL3 methyltransferase increased in gastric cancer tissues and paracancer tissues. (a) Methylated RNA (m6A) levels in 40 pairs of adult gastric cancer tissues and paracancer tissues. ^*∗∗*^*P* < 0.01. (b) mRNA levels of m6A-modified enzymes (METTL3, WTAP, METTL14, VIRMA, RBM15, FTO, and ALKBH5) were analyzed by qPCR. ^*∗*^*P* < 0.05. (c) Western blot analysis was used to detect the expression level of METTL3 protein in gastric cancer tissues and adjacent tissues. ^*∗*^*P* < 0.05. (d) Levels of methylated RNA (m6A) in human gastric cancer cell lines (AGS, MKN-45, and MKN-28) and normal gastric mucosal epithelial cells (GES-1). ^*∗*^*P* < 0.05. (e) mRNA levels of METTL3 in human gastric cancer cell lines (AGS, MKN-45, and MKN-28) and normal gastric mucosal epithelial cells (GES-1) were analyzed by qPCR. ^*∗*^*P* < 0.05. (f) Western blot analysis detected the expression level of METTL3 protein in human gastric cancer cell lines (AGS, MKN-45, and MKN-28) and normal gastric mucosal epithelial cells (GES-1).

A total of 40 pairs of fresh gastric cancer tissues and corresponding adjacent tissues were collected, and the total methylated RNA (m6A) levels were analyzed to explore the role of m6A in gastric cancer (Figure 1(a)). Compared with adjacent tissues, the expression of m6A was significantly higher in gastric cancer tissues than in normal tissues. The detection of mRNA levels of m6A-modified enzymes (METTL3, WTAP, METTL14, VIRMA, RBM15, FTO, and ALKBH5) in gastric cancer tissues and adjacent tissues showed that the mRNA level of METTL3 was significantly higher in gastric cancer tissues. Also, the mRNA level of ALKBH5 was higher in gastric cancer tissues than in adjacent tissues, while the mRNA level of FTO was lower in gastric cancer tissues than in adjacent tissues (Figure 1(b)). The expression of METTL3 mRNA in gastric cancer tissues was significantly different from that in adjacent tissues. Therefore, immunoblotting experiments were conducted, revealing that the level of METTL3 protein was significantly higher in gastric cancer tissues than in adjacent tissues (Figure 1(c)). Compared with the normal gastric mucosal epithelial cell line (GES-1), the level of m6A in the human gastric cancer cell lines (AGS, MKN-45, and MKN-28) increased to different degrees (Figure 1(d)). The mRNA level of METTL3 also increased to different degrees compared with the level in the normal cell line (Figure 1(e)). The level of METTL3 protein in the gastric cancer cell line was also significantly different from that in the normal cell line (Figure 1(f)). These results indicated the involvement of METTL3 in promoting human gastric cancer through m6A modification.

### 3.2. Effects of the Overexpression and Knockdown of the *METTL3* Gene on the Proliferation, Migration, and Invasion of Gastric Cancer Cells *In Vitro*

**Figure 2:**
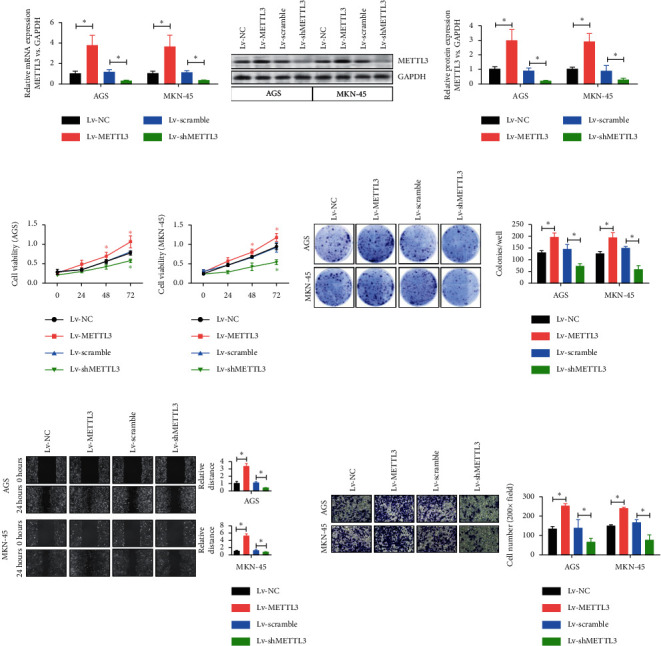
METTL3 silencing inhibited the proliferation, migration, and invasion of gastric cancer cells *in vitro*. (a) Silencing efficiency of METTL3 in AGS and mKN-45 cells was detected by qPCR. ^*∗*^*P* < 0.05. (b) Silencing efficiency of METTL3 in AGS and mKN-45 cells was detected by western blot analysis. ^*∗*^*P* < 0.05. (c) Proliferation levels of AGS and mKN-45 cell lines were determined and analyzed by CCK-8. ^*∗*^*P* < 0.05. (d) Colony formation rates of AGS and mKN-45 cell lines were detected by the colony formation assay. ^*∗*^*P* < 0.05. (e) Migration ability of AGS and mKN-45 cell lines was detected by cell scratch assay. ^*∗*^*P* < 0.05. (f) Invasion ability of AGS and mKN-45 cell lines was detected by Transwell assay. ^*∗*^*P* < 0.05.

Human gastric cancer cells (AGS and MKN-45) were selected as observation objects, and lentiviral-mediated shRNA was used to silence the expression of METTL3 to further explore the effect of METTL3 on the proliferation, migration, and invasion of gastric cancer cells *in vitro*. The silencing efficiency of METTL3 in AGS and MKN-45 cells was confirmed by quantitative PCR (Figure 2(a)) and western blot analysis (Figure 2(b)). Then, cell proliferation tests were performed on human gastric cancer cell lines AGS and MKN-45, revealing that the cell proliferation of both cell lines increased gradually and the amplitude of this proliferation decreased with the silencing of METTL3 (Figure 2(c)). Subsequently, colony formation experiments were conducted. Both AGS and MKN-45 cell lines showed that the group with overexpression of the *METTL3* gene had a higher colony formation rate compared with the group with the deletion of the *METTL3* gene. The group with *METTL3* gene knockout had a lower cell colony formation rate compared with the control group (Figure 2(d)). Finally, the migration and invasion abilities of gastric cancer cell lines were explored. The results showed that METTL3 silencing significantly inhibited migration (Figure 2(e)) and invasion ability (Figure 2(f)) in AGS and MNK-45 cell lines. These results indicated that the silencing of METTL3 inhibited the proliferation, migration, and invasion abilities of gastric cancer cells *in vitro*.

### 3.3. Effects of the Overexpression and Knockdown of the *METTL3* Gene on the YAP Signaling Pathway in Gastric Cancer Cells *In Vitro*

**Figure 3:**
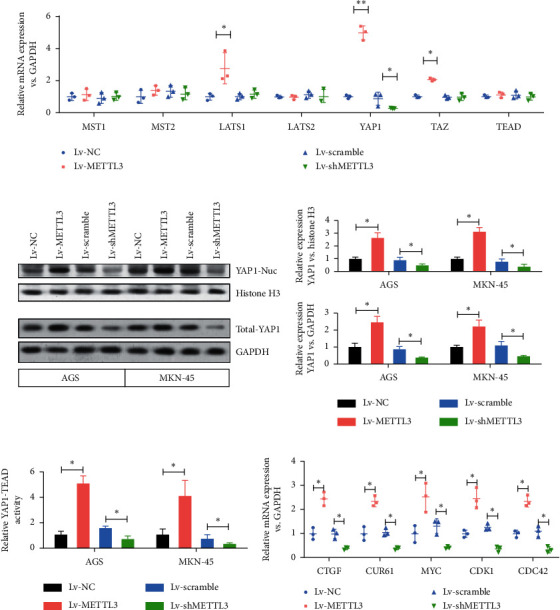
METTL3 silencing inhibited both the expression of YAP1 and the activity of the YAP1 signaling pathway. (a) Expression level of YAP signaling pathway-related genes (MST1/2, LATS1/2, YAP1, TAZ, and TEAD) was detected by qPCR. ^*∗*^*P* < 0.05,^*∗∗*^*P* < 0.01. (b) The YAP1 nucleoprotein and total YAP1 protein were detected by western blot analysis following the silencing of the *METTL3* gene. ^*∗*^*P* < 0.05. (c) Activity of the YAP1 in nuclear components was detected by TEAD-dependent luciferase activity. ^*∗*^*P* < 0.05. (d) After YAP1 is combined with TEAD, the transcriptional activation effect on typical downstream genes of the YAP1 signaling pathway following the silencing of *METTL3*. ^*∗*^*P* < 0.05.

This study analyzed whether the downstream component of the Hippo signaling pathway, YAP1, was involved in human gastric cancer to explore the mechanisms of *METTL3*-silencing-mediated tumor inhibition. The expression levels of YAP signaling pathway-related genes (*MST1/2*, *LATS1/2*, *YAP1*, *TAZ*, and *TEAD*) mRNA were detected, revealing that *METTL3* silencing significantly reduced the expression levels of YAP1 mRNA (Figure 3(a)). Then, we detected the changes in YAP1 nucleoprotein and total YAP1 protein following the silencing of the *METTL3* gene. We found that the expression of YAP1 nucleoprotein and total YAP1 protein decreased compared with the control group following the silencing of *METTL3* (Figure 3(b)) in AGS and MKN-45 cells. After YAP1 is combined with TEAD, the activity of YAP1, a downstream target gene of *METTL3*, is further evaluated. The silencing of *METTL3* significantly reduced the activity of the YAP1-TEAD in AGS and MKN-45 cell lines (Figure 3(c)). After YAP1 is combined with TEAD, we detect the transcriptional activation effect on typical downstream genes of the YAP1 signaling pathway following the silencing of *METTL3*. We found *METTL3* silencing can significantly inhibit typical downstream target genes of YAP1 (Figure 3(d)). These results indicated that the silencing of *METTL3* suppressed the expression of YAP1 and it also inhibited the activity of the YAP1 signaling pathway *in vitro*.

### 3.4. METTL3 Affected the Expression of YAP1 by Regulating the m6A Modification Level of the *YAP1* Gene

**Figure 4:**
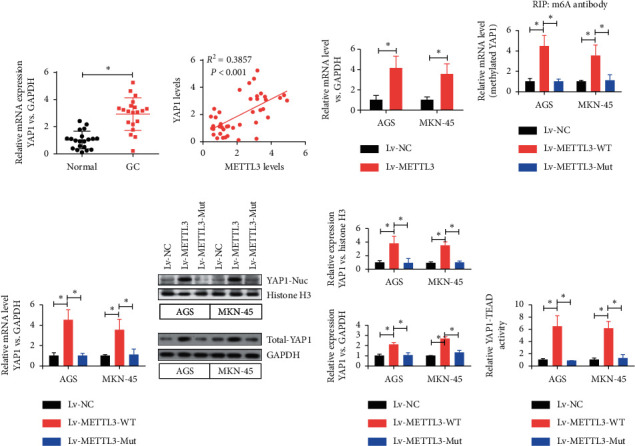
METTL3 regulated the expression level of the *YAP1* gene in AGS and MKN-45 cells through m6A methylation modification. (a) Detection of YAP1 mRNA levels in gastric cancer tissues and normal tissues by qPCR. ^*∗*^*P* < 0.05. (b) Linear regression analysis was used to evaluate the positive correlation between the expression of *METTL3* and *YAP1*. *R*^2^ = 0.3857, *P* < 0.001. (c) Relationship between the expression of the *METTL3* and mRNA level of YAP1 was detected by qPCR. ^*∗*^*P* < 0.05. (d) Effect of the overexpression and mutation of *METTL3* on the m6A level of YAP1 was detected by the m6A RIP experiment. ^*∗*^*P* < 0.05. (e) Overexpression of *METTL3* and the relationship between the mutation and the mRNA level of YAP1 were detected by qPCR. ^*∗*^*P* < 0.05. (f) Relationship between the overexpression and mutation of *METTL3* and YAP1-related protein levels was detected by western blot analysis. ^*∗*^*P* < 0.05. (g) Relationship between the overexpression and mutation of *METTL3* and the activity of the YAP1 signaling pathway. ^*∗*^*P* < 0.05.

The mRNA levels of YAP1 in gastric cancer tissues and normal tissues were detected to further investigate the role of the METTL3-mediated m6A modification of the *YAP1* gene in gastric cancer, revealing that the mRNA levels of *YAP1* were significantly higher in gastric cancer tissues than in normal tissues (Figure 4(a)). The linear regression analysis showed that the YAP1 level positively correlated with the METTL3 level in human gastric cancer tissues (Figure 4(b)). The qPCR analysis showed that *METTL3* significantly increased the YAP1 mRNA levels in AGS and MKN-45 cell lines (Figure 4(c)). RIP experiments found that the overexpression of *METTL3* significantly increased the m6A level of YAP1 (Figure 4(d)) and also increased the expression levels of YAP1 mRNA (Figure 4(e)), YAP1 nucleoprotein, and total YAP1 protein (Figure 4(f)) in AGS and MKN-45 cell lines. On the contrary, by constructing mutants with the deletion of METTL3 enzyme activity, it was found that the mutant *METTL3* showed the loss of elevated m6A levels of YAP1 (Figure 4(d)) and also reduced levels of YAP1 mRNA (Figure 4(e)), YAP1 nucleoprotein, and total YAP1 protein (Figure 4(f)). The results of TEAD-dependent luciferase activity showed that the overexpression of *METTL3* significantly increased the activity of the YAP1 signaling pathway compared with the control group and METTL3 enzyme-deficient mutants (Figure 4(g)). The results showed that METTL3 increased the expression level of the *YAP1* gene in AGS and MKN-45 cells through m6A methylation modification.

### 3.5. Inhibitory Effect of Peptide 17 on the YAP1 Signaling Pathway Eliminated the Promoting Effect of *METTL3* on the Proliferation and Migration of Gastric Cancer Cells

**Figure 5:**
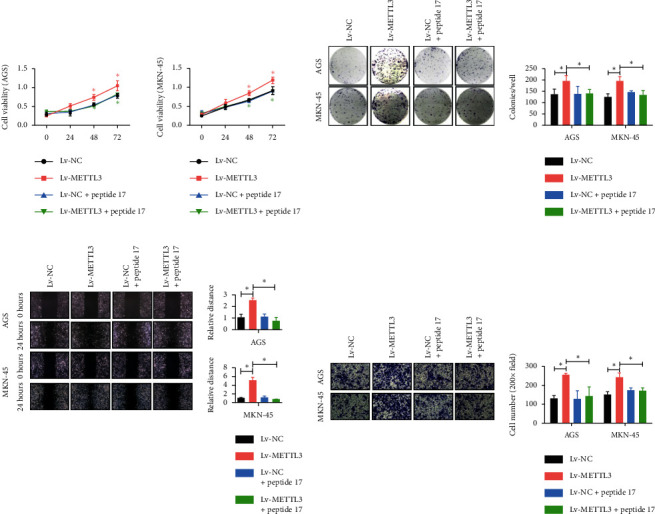
Inhibitory effect of peptide 17 on the YAP1 signaling pathway eliminated the promoting effect of *METTL3* on the proliferation and migration of gastric cancer cells. (a) Proliferation levels of AGS and MKN-45 cell lines were determined and analyzed by CCK-8. ^*∗*^*P* < 0.05. (b) Colony formation capacity of AGS and mKN-45 cell lines was detected by the colony formation assay. ^*∗*^*P* < 0.05. (c) Migration ability of AGS and MKN-45 cell lines was detected by the cell scratch assay. ^*∗*^*P* < 0.05. (d) Invasion ability of AGS and mKN-45 cell lines was detected by the transwell assay. ^*∗*^*P* < 0.05.

The study further examined the effect of *YAP1* on the proliferation of GC cells and the biological function of migrating cells caused by the overexpression of the *METTL3* gene. YAP-TEAD inhibitor 1 (peptide 17) was used to suppress the expression of *METTL3*, and the silencing of METTL3 was detected in cell lines AGS and MKN-45. The cell proliferation levels of the two cell lines showed different degrees of decline (Figure 5(a)). Subsequently, colony formation assay was used to detect the significantly higher number of gastric cancer cells containing *METTL3* in AGS and MKN-45 cell lines than in the *METTL3* gene silencing and the other two control groups (Figure 5(b)). The cell scratch assay and Transwell assay revealed that the migration and invasion of the *METTL3* gene were significantly enhanced in AGS and MKN-45 cell lines compared with knockdown METTL3 (Figures 5(c) and 5(d)). These results confirmed that the inhibition of the YAP1 signaling pathway eliminated the promoting effect of *METTL3* on the proliferation and migration of gastric cancer cells.

### 3.6. *METTL3* Overexpression in the Subcutaneous Tumor Model of Nude Mice Can Lead to Tumor Growth

**Figure 6:**
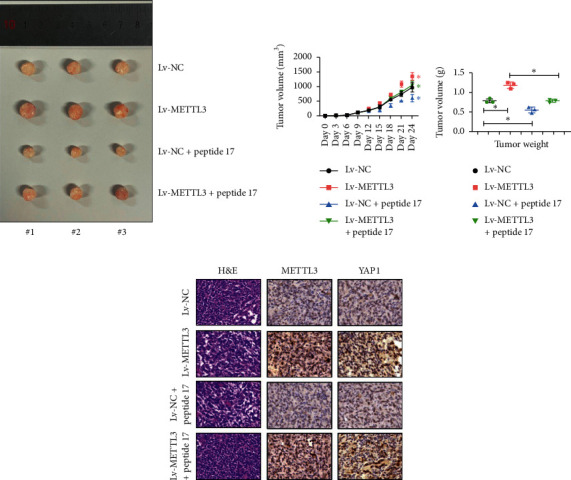
*METTL3* overexpression in the subcutaneous tumor model of nude mice can lead to tumor growth. (a) Tumor size was detected in the subcutaneous tumor formation model of nude mice. (b) Tumor volume was measured in the subcutaneous tumor formation model of nude mice. ^*∗*^*P* < 0.05. (c) Tumor weight was measured in the subcutaneous tumor formation model of nude mice. ^*∗*^*P* < 0.05. (d) Expression of METTL3 and YAP1 in tumor tissues was detected by immunohistochemistry.

Finally, a subcutaneous tumor model of nude mice was established. The tumor growth was significant in mice overexpressing the *METTL3* gene compared with the control group (Figure 6(a)). The tumor volume gradually increased with time and was significantly higher than that in the other control groups on days 18, 21, and 24 (Figure 6(b)); the tumor weight also increased significantly (Figure 6(c)). Subsequently, the expression of *METTL3* and *YAP1* in tumor tissues was detected by immunohistochemistry, revealing that the expression level of *METTL3* in tumor tissues positively correlated with the expression level of *YAP1*; the expression level of METTL3 also decreased after the YAP1 signaling pathway was inhibited by peptide 17 (Figure 6(d)). These results further proved that the inhibition of YAP1 activity significantly reversed the promoting effect of the overexpression of *METTL3* on gastric cancer cells in mice.

## 4. Discussion

Modification of m6A is the most common chemical modification of human mRNA [25], which can affect the complexity of cancer progression by regulating biological functions associated with cancer [10]. *METTL3* is a key component of the m6A methyltransferase complex. The increase of *METTL3* is related to the poor prognosis of patients with gastric cancer [26, 27]. However, the specific mechanism of *METTL3* in the proliferation and metastasis of gastric cancer is still unclear. This study showed that m6A methyltransferase *METTL3* promoted the proliferation and metastasis of gastric cancer through the modification of YAP1 mRNA m6A.

The present study found that the levels of m6A and METTL3 were significantly higher in gastric cancer tissues and gastric cancer cell lines compared with normal paracancer tissues. Therefore, the *METTL3* gene was knocked out. After the knocking out of *METTL3*, the activity of the YAP1 signaling pathway and the proliferation and metastasis of gastric cancer were inhibited by regulating the m6A modification level of the *YAP1* gene. In addition, this study further found that the inhibition of the expression of the YAP1 signaling pathway by peptide 17 *in vitro* and nude mice tumor formation models also eliminated the promoting effect of *METTL3* on the proliferation and metastasis of gastric cancer cells. This study showed that *YAP1* was a downstream target of *METTL3*. The methylation level of YAP1 mRNA was reduced in gastric cancer cells inhibited by *METTL14*. These data further proved that the role of METTL3 in the development of gastric cancer was dependent on the methylation and activation of YAP1 and YAP1 signaling pathways, further confirming that m6A methyltransferase METTL3 promoted the proliferation and metastasis of gastric cancer by modifying YAP1 mRNA m6A. It is worth mentioning that LATs1 is a tumor suppressor that can promote the activation of Hippo signaling [28]. But, in Figure 3(a), the expression level of LATS1 is significantly increased after METTL3 is overexpressed, but the increase ratio was far less than that of YAP1. We speculate that the increase in LATS1 expression might be a negative feedback regulation of YAP1 pathway activation and the inhibitory effect of the increase of LATS1 expression on YAP1. LATS1 may be offset by increased YAP1 expression. On the other hand, we can see that the downregulation of METTL3 has no significant effect on the expression level of LATS1, but significantly inhibits the expression of YAP1; therefore, we speculate that the upregulation and activation of YAP1 by METTL3 is the main one.

YAP1, as a downstream target of METTL3, is essential for regulating the proliferation and differentiation of various adult cells [29]. YAP1 is a core effector in the Hippo pathway and has been identified as a key regulator of tissue homeostasis and organ development [22], which can be phosphorylated and inactivated by Hippo phosphorylated protein kinase Warts (Wts) [23]. The Hippo pathway was first discovered in *Drosophila*. Hippo pathways are also found in mammals; they participate in tumorigenesis by regulating the tumor microenvironment and adaptive immune response [30]. Studies have shown that YAP1 can recruit M2 macrophages, bone-marrow-derived suppressor cells, and regulatory T cells, thereby inhibiting host-effector T cells in the tumor microenvironment and further leading to cancer progression and drug resistance [31]. Some scholars collected 156 gastric cancer samples and found that 48.7% (76/156) of patients with gastric cancer showed a low expression level of YAP1, while 51.3% (80/156) of the patients with gastric cancer showed a high expression level of YAP1. The results of a 20-year follow-up revealed that YAP1 was associated with poor overall survival [32].

## 5. Conclusions

In conclusion, this study showed that m6A methyltransferase *METTL3* promoted the proliferation and metastasis of gastric cancer through the m6A modification of the YAP1 pathway. The discovery of the METTL3-YAP1 pathway provided a new direction for the treatment of gastric cancer. The discovery of the METTL3-YAP1 pathway also provided a new target for the treatment of gastric cancer.

## Figures and Tables

**Table 1:** The primers in qPCR.

Gene	Forward primer (5′–3′)	Reverse primer (5′–3′)
METTL3	CAAGCTGCACTTCAGACGAA-	GCTTGGCGTGTGGTCTTT
METTL14	CTACCCATCCTCACTGTCAGTC	GGATGTTCCTGTTTGACCTGAGG
RBM15	TCCCACCTTGTGAGTTCTCC	GTCAGCGCCAAGTTTTCTCT
WTAP	CTTCCCAAGAAGGTTCGATTGA	TCAGACTCTCTTAGGCCAGTTAC
VIRMA	AATCCTGTGGGAAGATCAGC	ACACGTAAGGCAGTGGTAAG
FTO	CCAGAACCTGAGGAGAGAATGG	CGATGTCTGTGAGGTCAAACGG
ALKBH	CCAGCTATGCTTCAGATCGCCT	GGTTCTCTTCCTTGTCCATCTCC
MST1	CACCGATTTACGCCAGAAAA	GAAGTTCTCCTCCAGTTGTG
MST2	ACCACAACCAACCTCCATTC	CTTGGCCATCAAATTCAGTC
LATS1	CCACCCTACCCAAAACATCT	TCACTCTCATCTTCCTTGGG
LATS2	GTGGACCTGTATGAATTGGG	TGGTGGCTGTTGAAGGAGTT
YAP1	ATGAGATGGATACAGGTGATA	TAAGGATGTCAGAACTCAAAG
TAZ	AGAGAATGAGGGGAAAGGTG	AGGATGATGGGGTTGAGATG
TEAD	TCAAGCCTTTTGTGCAGCAG	GCCCCAGCATCATCTTGAAT
GAPDH	TGACTTCAACAGCGACACCCA	CACCCTGTTGCTGTAGCCAAA

## Data Availability

The experimental data used to support the findings of this study are available from the corresponding author upon request.
